# Aquifer Potential Assessment in Termites Manifested Locales Using Geo-Electrical and Surface Hydraulic Measurement Parameters

**DOI:** 10.3390/s19092107

**Published:** 2019-05-07

**Authors:** Jamilu Bala Ahmed II, Biswajeet Pradhan, Shattri Mansor, Zainuddin M. Yusoff, Salamatu Abraham Ekpo

**Affiliations:** 1 Department of Civil Engineering, Faculty of Engineering, Universiti Putra Malaysia, Serdang 43400, Malaysia; senjamil32@gmail.com (J.B.A.II); shattri@upm.edu.my (S.M.); zmy@upm.edu.my (Z.M.Y.); 2 Department of Geology, Faculty of Science, Federal University Lokoja, 1154 Lokoja, Nigeria; 3 Centre for Advanced Modelling and Geospatial Information Systems (CAMGIS), Faculty of Engineering and Information Technology, University of Technology Sydney, Sydney, NSW 2007, Australia; 4 Department of Energy and Mineral Resources Engineering, Choongmu-gwan, Sejong University, 209 Neungdong-ro Gwangjin-gu, Seoul 05006, Korea; 5 Department of Environmental Management, Faculty of Environmental Science, Nasarawa State University, 1022 Keffi, Nigeria; abrahamsalamatu@gmail.com

**Keywords:** termite mounds, groundwater, infiltration, VES, GIS, Nigeria

## Abstract

In some parts of tropical Africa, termite mound locations are traditionally used to site groundwater structures mainly in the form of hand-dug wells with high success rates. However, the scientific rationale behind the use of mounds as prospective sites for locating groundwater structures has not been thoroughly investigated. In this paper, locations and structural features of termite mounds were mapped with the aim of determining the aquifer potential beneath termite mounds and comparing the same with adjacent areas, 10 m away. Soil and species sampling, field surveys and laboratory analyses to obtain data on physical, hydraulic and geo-electrical parameters from termite mounds and adjacent control areas followed. The physical and hydraulic measurements demonstrated relatively higher infiltration rates and lower soil water content on mound soils compared with the surrounding areas. To assess the aquifer potential, vertical electrical soundings were conducted on 28 termite mounds sites and adjacent control areas. Three (3) important parameters were assessed to compute potential weights for each Vertical Electrical Sounding (VES) point: Depth to bedrock, aquifer layer resistivity and fresh/fractured bedrock resistivity. These weights were then compared between those of termite mound sites and those from control areas. The result revealed that about 43% of mound sites have greater aquifer potential compared to the surrounding areas, whereas 28.5% of mounds have equal and lower potentials compared with the surrounding areas. The study concludes that termite mounds locations are suitable spots for groundwater prospecting owing to the deeper regolith layer beneath them which suggests that termites either have the ability to locate places with a deeper weathering horizon or are themselves agents of biological weathering. Further studies to check how representative our study area is of other areas with similar termite activities are recommended.

## 1. Introduction

Termites are among the most emphatic and important soil organisms in tropical ecosystems [1,2,3]. Their nests (also called mounds) are constructed from surrounding soils but differ from the surrounding soil matrix in terms of physical and chemical compositions due to termite reworking of the soils to modify its porosity, water infiltration capacity, and pH level, among others [4,5,6,7]. These mounds often materialize into islands of enhanced soil water availability to support lush vegetation in dry periods or environments [8,9]. This is similar to vegetation lineaments where lush vegetation follows the surface traces of faults, rock boundaries (such as dyke intrusion into country rock) and valleys along stream channels as a result of the wetter subterranean conditions in those environments [10]. Fault zones, fractured dykes and valleys are excellent localities for groundwater prospecting as they serve as conduits through which groundwater preferentially flows [11]. 

Apart from flourishing vegetation around mounds, there is other evidence from the field and literature suggesting a relationship between termite mounds and groundwater (e.g., [8,12,13,14]). These studies have been discussed by Ahmed II and Pradhan [15] to include wet soils on termite mounds during periods of mound rebuilding, which is usually done in the dry season, and assemblages of mounds near groundwater outcrops (seep zones). Other examples reported include lining up of mounds on fracture dense dykes that serve as conduits for groundwater transport [16], preferential flow of water along macro-pores created by termites bypassing the soil matrix to enhance groundwater recharge [17], higher infiltration rates compared with adjacent areas, and the observance of some species of termites burrowing down to water table depths to access groundwater for body metabolism [18]. Despite these significant clues, studies to test the prospective groundwater around termite mounds for possible community-based supply are presently unavailable. It is common knowledge that people living in tropical to sub-tropical areas of the world (where termite activities are more perceptible) are amongst the poorest [19], largely due to water shortages that impacts on their health, education, income and social inclusion [20,21]. 

Few studies have suggested that termite mounds are likely biomarkers of groundwater [16,17,22], even though the potential has yet to attract the interest of researchers in the field of groundwater hydrology. Discovering whether all termite mounds indicate the occurrence of groundwater at shallow depth within their immediate environments or it is only specific mound types (in terms of their physical features and/or inhabitant species) would further strengthen our understanding of the relationship that exists between termites and groundwater. Groundwater investigations around termite mounds are unconventional, so there is a lack of reference material in the hydro-geological literature. This study is the first of its kind to prospect for groundwater around termite mounds with the main aim of assessing the aquifer potential beneath termite mounds and comparing it with adjacent control areas.

## 2. Materials and Methods

### 2.1. Geography and Geological Setting of the Study Area

**Figure 1:**
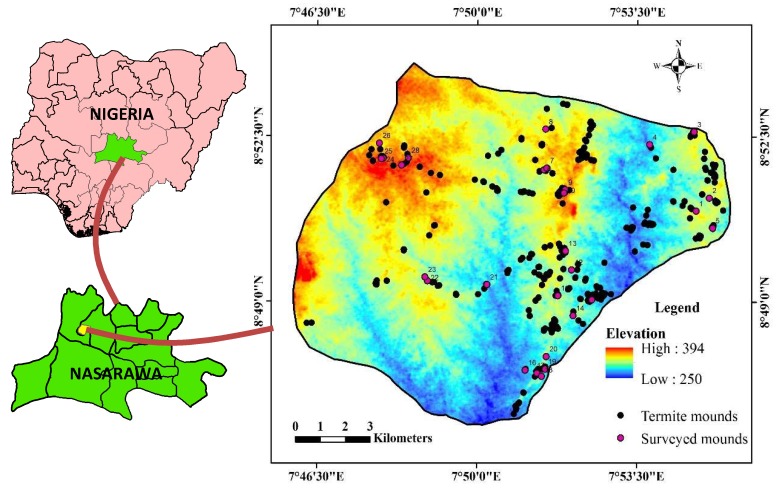
Digital elevation model (DEM) of study site showing all the mapped termite mounds (black dots) and the surveyed termite mounds (pink dots).

The study area is an ancient town in central Nigeria called Keffi. It lies between latitude N8°46′40″–N8°53′30″ and longitude E7°46′03″–E7°55′30″, covering an area of about 156 km^2^ (Figure 1). The area is characterized by a tropical climatic condition with distinct wet and dry periods [23]. The annual rainfall which falls between May to October ranges between 850–1800 mm and the annual temperature range is between 22–34 °C. The vegetation is typically that of the Guinea Savannah belt that extends from western Senegal to eastern Nigeria, but has largely been altered due to human activities [24]. The main land use in the area is residential and agricultural [25], where tuber (e.g., *Dioscorea alata* and *Ipomoea batatas*), leguminous (e.g., *Phaseolus vulgaris* and *Arachis hypogaea*) and cereal (e.g., *Zea mays* and *Oryza sativa*) crops are grown.

The topography is undulating with elevation ranging from 250 m to 394 m above mean sea level. The highest point is at Captain Maloney Hill in the central part of the town that sits on a gneissic ridge. A dendritic pattern with a southerly orientation characterize the drainage system, with Uke and Antau as the major stream channels whose flow directions are concordant with the structural trend of the underlying crystalline basement rocks [26]. In terms of geology, the study area is situated within the basement complex of central Nigeria with three major distinguishable lithological units, namely, the Precambrian migmatitic gneisses, Upper Proterozoic schist and Lower Paleozoic older granites [27,28], with sporadic occurrences of pegmatites and quartz dykes. As with many basement complex environments, groundwater occurrence is highly variable and occurs only in the weathered and fractured bedrock aquifers [29,30,31]. Aquifers in this region are recharged by direct rainfall infiltration and infiltration through stream and lateral subsurface flows [32]. Groundwater is a major source of clean water for the inhabitants of the area since the piped water supply had become grossly inadequate. Groundwater has several advantages over surface water which include; availability at point of need, little or no treatment required, less susceptible to drying up and less vulnerable to catastrophic events [33,34,35].

### 2.2. Methods

A multidisciplinary methodology was adopted to achieve the stated objectives, and this was carried out in four stages: (1) Field mapping and inventory of termite mounds across the study site, recording their coordinates using hand-held GPS (accuracy ±3 m) and physical features, which included mound height, mound basal diameter, mound activity status, and whether the mound was under a tree canopy or not. (2) The field experiment and sampling stage encompassed field infiltration measurements, soil and termite species sampling for analyses of particle size distribution, water content, bulk density, porosity on mounds and control areas, as well as termite species identification. The analyzed parameters at this stage were compared with those of adjacent control areas which were 10 m away from each mound. (3) The hydro-geophysical stage involved the use of electrical resistivity measurements to acquire resistivity data, processing and interpreting those data to determine primary geo-electrical parameters beneath termite mounds and control areas. (4) The analysis stage involved the assessment of aquifer potential using three geo-electrical parameters. The inventory of the surveyed termite mounds, which is also the site of soil sampling, infiltration test and resistivity measurement, are shown in Figure 1 and Table 1. Details of the four stages are highlighted in the following subsections.

#### 2.2.1. Field Mapping of Termite Mounds

A ground-based mapping approach was adopted to cover the 156 km^2^ study area. The area was traversed on foot along 2 major highways and 66 minor roads and footpaths, which aided the mapping and recording of 361 termite mounds locations together with their physical characteristics using Survey 123 for ArcGIS mobile application. From the 361 mapped termite mounds, six cluster zones of the mounds were drawn. From each of five clusters, five mounds were selected, while only three were selected in the sixth cluster, resulting in a total sample of 28 mounds. Further field experiments and soil sampling measurements were taken from these 28 termite mounds.

#### 2.2.2. Field Sampling and Experiment

##### Soil Texture

**Figure 2:**
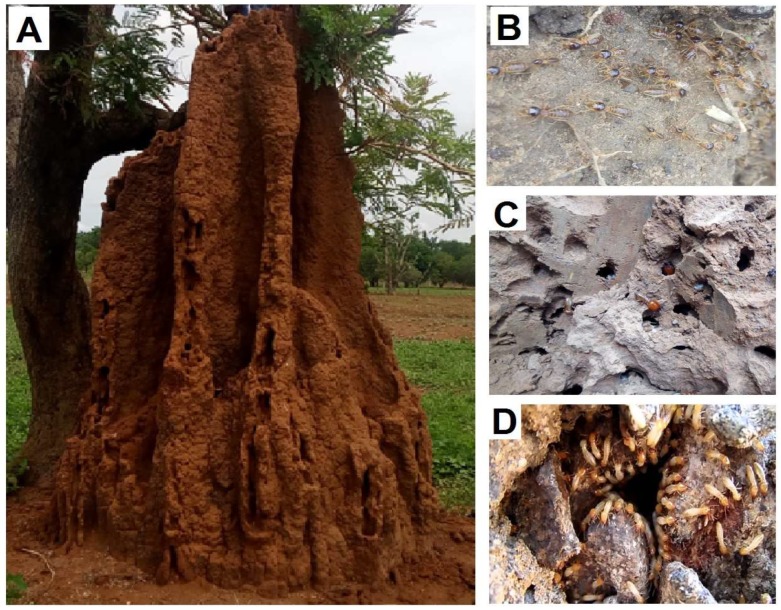
Termite mound and live termite species encountered in the study area. (**A**) Cathedral shaped termite mound, (**B**) *Nasutitermes* species, (**C**) *Macrotermes* species, (**D**) *Coptotermes* species.

Soil sample collection was conducted in two forms: On termite mounds and away from mounds as control. Disturbed soil samples were taken at 10 m radius away from mounds at a depth of 0–20 cm from the surface to form the first group of composite samples (control). For the second group, on termite mounds (Figure 2), soils were collected from the top, middle and foot of the mounds at 0–20 cm depth to form the composite samples. The samples were air dried, ground, and allowed to pass through a 2 mm sieve, and then were kept for further analysis. Particle size (i.e., % sand, silt and clay) was determined by Bouyoucos hydrometer method [36]. This method is based on Stokes’ Law which corroborated a relationship between the sizes of particles and their rate of sedimentation in a water solution [36].

##### Bulk Density and Porosity

Soil samples were collected from termite mounds and control sites at 0–10 cm depth using core samplers 2.6 cm in diameter and 10 cm long. The samples were oven dried at 105 °C for 24 h, after which the dry bulk density and soil porosity were calculated using Equations (1) and (2):

(1) $$\mathrm{Bulk}\;\mathrm{density}\;\left(g/cm^{3}\right)=\frac{Weigh\;of\;dry\;bulk\;sample}{Volume\;of\;soil\;core\;\left(cm^{3}\right)}$$

(2) $$\mathrm{Total}\;\mathrm{porosity}\;\left(\%\right)=1-\frac{Bulk\;density\;\left(g/cm^{3}\right)}{2.65}$$

where 2.65 is the soil particle density (assumed to be constant).

##### Soil Water Content

The surface 0–10 cm core samples obtained were first weighed before oven drying. They were reweighed after 24 h of drying to examine the percentage water content in the soils using Equation (3):

(3) $$\mathrm{Soil}\;\mathrm{water}\;\mathrm{content}\;\left(\%\right)=\frac{Weight\;of\;wet\;soil\;-\;Weight\;of\;dry\;soil}{Weight\;of\;dry\;soil}\times \frac{100}{1}$$

##### Infiltration Rate

The infiltration rate on mounds and control soils was measured using the falling head method. A 7.62 cm diameter steel cylinder was buried 10 cm into the soil and at termite mound surfaces. An inch (107 mL) of distilled water was carefully added into the ring and the time taken for the water to drain was recorded. This was repeated 3 times each, and the average of the readings was recorded.

#### 2.2.3. Hydro-Geophysical Survey

**Figure 3:**
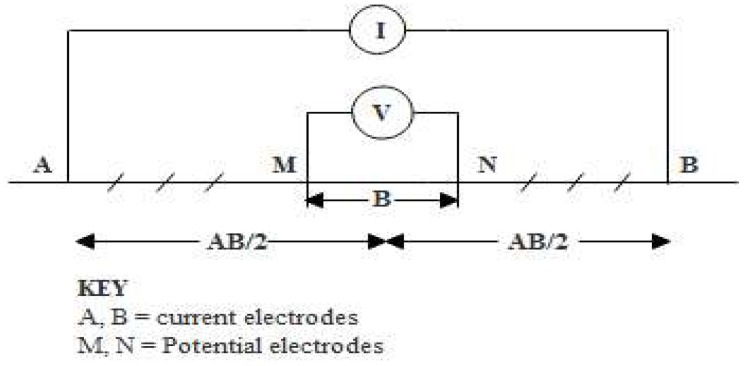
A schematic representation of VES layout using the Schlumberger electrode array, modified from [52].

To assess the subsurface layer responses to electrical current, determine the nature and composition of the concealed subsurface formation, and thus, the aquifer potential beneath termite mounds and control areas, vertical electrical soundings (VES) data were acquired from each of the 28 mound locations and control points. VES is an electrical resistivity method that involves passing electrical current into the ground through a pair of electrodes and recording the potential difference from another pair of electrodes (Figure 3). It is used to determine the depth, thickness, boundary and yield potential of an aquifer. Electrical resistivity measurements involving VES is one of the most important geophysical technique employed in many regions for groundwater investigation owing to its cost effectiveness and ability to probe deeper parts of the sub-surface [37,38]. Consequently, it has found extensive application in the fields of groundwater hydrology [39,40,41], geotechnical engineering [42,43,44], agriculture [45] and environmental studies [46,47]. This, therefore, constitutes the basis for the choice of VES survey in this study.

From the established 56 stations, the ABEM SAS 1000 resistivity meter using the Schlumberger configuration as described by [48] was deployed to inject electrical current into the ground through two current electrodes (AB) and the difference in potential measured by two other potential electrodes (MN) (Figure 3). The desired depth of investigation largely depends on the distance between the current electrodes separation [49,50]. As such, a maximum current electrode separation (AB) of 200 m was adopted, whereas potential electrodes separation (MN) varied intermittently between 0.5–10 m to enable a suitable depth penetration that invariably ensured appropriate delineation of the subsurface formation. From the obtained current (I) and voltage (V) readings, apparent resistivity (*ρa*) was calculated using Equation (4):

(4) $$\rho a=K\frac{V}{I}$$

where *K* is a geometric factor and is dependent on electrodes arrangement. *K* can be given as

(5) $$K=\pi \;\left(\frac{AB^{2}}{2}\right)-\left(\frac{MN^{2}}{2}\right)/MN$$

where *AB*^2^ is half current electrode separation and *MN*^2^ is half potential electrode separation. Resistivity R is given by

(6) $$R=\frac{V}{I}$$

Therefore, Equation (4) (to calculate apparent resistivity (*ρa*)) can be substituted by

(7) ρa=KR

Further, the obtained apparent resistivity readings at each station were plotted against the corresponding current electrode separation (AB/2) on log–log graph sheets from where initial resistivity curves were generated using information from the geology and well litho-logs to reduce ambiguity in the interpretation. The VES curves provided information on the vertical variations in the resistivity of the ground with depth. A 1-D inversion computer iteration program, EarthImager, produced by Advanced Geosciences Incorporation (AGI), was then engaged to quantitatively interpret model curves by producing the best fit between the field curves and calculated ones. A model curve is only acceptable when the root mean square error (RMSE) is ≤10% [51]. Hence, the layer resistivity and thicknesses were estimated from the interpreted models.

#### 2.2.4. Aquifer Potential Assessment

Geo-electrical parameters derived from VES have proved to be valuable in the preliminary assessment of aquifer potentials (e.g., [38,40,50]). A scheme developed by Olayinka [53] has gained popularity [26,29,54] and was used here to assess the aquifer potential beneath termite mounds and corresponding control areas. The scheme takes into consideration three primary geo-electrical parameters, namely, depth to bedrock (regolith thickness), weathered bedrock (saprolite) resistivity, and fresh/fractured bedrock (saprock) resistivity, and assigns weights ranging from 2.5 (least suitable class in a factor) to a maximum of 10 (most suitable class in a factor). A geometric mean is then computed from where values between 0–5, 5–7 and 7–10, representative of low, medium and high potentials, respectively. Table 2 summarizes the weighting criteria of the scheme. Furthermore, a map of aquifer potential of the study area was prepared from the computed geometric mean of aquifer potential beneath termite mounds using the Inverse Distance Weight (IDW) interpolation procedure in the ArcGIS 10.5 environment. The IDW interpolation was chosen because it is based on weighted distance average where the average cannot be greater than the highest nor less than the lowest input.

### 2.3. Statistical Analysis

To compare means of physical and hydraulic properties between termite mounds and control areas, an Independent *t*-Test was performed where sample means were normally distributed; otherwise, and where an appropriate transformation was not found, a Mann–Whitney U-test was performed. Relationships between variables were tested using the Spearman’s rank correlation. All statistical analyses were performed in IBM SPSS statistics 23.

## 3. Results and Discussion

### 3.1. Soil Physical and Hydraulic Characteristics

There was considerable difference between soil texture on termite mounds and adjacent control soils and this difference is statistically significant (Table 3). Control area soils in the study site displayed texture ranging between sandy clay loam and fine sand (Table S1, Figure S1), indicating high percentage of sand, an average of about 13.7% more than that on termite mounds. However, on termite mounds, there was a decrease in sand content and consequently an increase in silt and clay proportions. Termites have the ability of selecting smaller size fractions of soils in mound building [55,56]. The higher clay and silt content of the mounds soils relative to adjacent soils is in agreement with the documented selective translocation of fine materials by termites [57,58,59,60], but this will likely result in poor vertical drainage of surface water. However, the activeness of a mound plays an important role on the soil texture. From our result, about 56% of non-active mounds either indicated an increase in sand proportion or recorded similar values compared to that from adjacent soils. Once a mound becomes inactive, re-building processes cease, thereby exposing the mounds to leaching and erosion, where finer, clayey materials are easily washed away. On the other hand, when mounds are active, especially with the original builders, there is a continuous rebuilding and continuous maintenance of the mounds by the termite workers after degradation from rain-splash and other forms of erosion [61].

The mean bulk density on termite mounds approximates that of adjacent control soils at about 1.93 g/cm^3^ (Table S2). This resulted in a non-significant difference in the values of porosity on termite mounds and control areas (t = 0.394, *p* = 0.695). Similar result have been recorded by Ackerman et al. [62] in Amazonia, Brazil. Porosity refers to the ratio between pore volume and the total volume of a material [63] and is dependent on several factors, including texture and structure of the material [64]. The relatively higher clay content on termite mounds can bring about the increase in pore sizes but not permeability, which will give rise to poor drainage conditions. This situation can account for the area in the vicinity of termite mounds to have a higher soil water potential to enhance plant growth and overall productivity compared to adjacent surroundings [6,8,65].

There is a slight but statistically significant difference (t = –4.249, *p* < 0.001) in the water holding capacity of mounds and adjacent area soils, with the adjacent area soils holding about 11% more water than termite mounds soils (Table S3). This is lower than the 25% difference obtained by Ackerman et al. [62]. The lower soil water content can be attributed to the harvesting activities of termites that create networks of underground galleries connected to the surface by foraging macro-pores through which water incident on the surface can easily infiltrate and which also serve to capture overland flow [17,66,67,68].

The rate of water infiltration on termite mounds under a falling head of pressure is about two times greater than that on adjacent control soils (U = 88.0, *p* < 0.001) (Table S4). Several studies have obtained similar results from different parts of the world [4,7,62,69,70]. Eldridge [69] recorded infiltration rates on mounds of *Drepanotermes species* in semi-arid eastern Australia to be between four and ten folds higher relative to that of the surroundings, while Ackerman et al. [62] documented the same rates five to six folds higher. Lower soil moisture, together with a higher infiltration rate around termite mounds, is likely to enhance the recharge rate of underlying groundwater reservoirs and consequently present a more viable potential source of groundwater compared to surrounding areas. Places with high rates of infiltration have been suggested as suitable for groundwater augmentation through artificial recharge [71].

### 3.2. Geo-Electrical Characteristics

**Figure 4:**
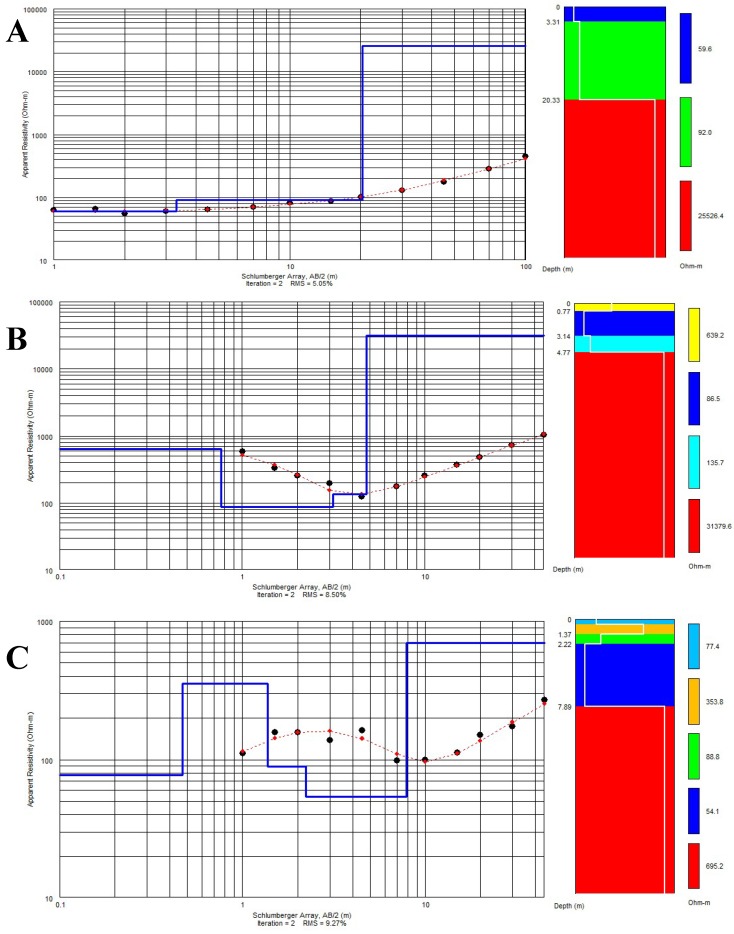
Samples of resistivity sounding curves obtained from the study site with their interpretations. (**A**) 3-layer curve model (**B**) 4-layer curve model (**C**) 5-layer curve model.

Qualitative interpretation of the VES model curves from termite mounds points revealed three to five geo-electric layer conditions (Figure 4). The characteristics of each of these layers are presented below and summarized in Table 4.

#### 3.2.1. Three-Layer Model

The first-layer of this model is composed of highly weathered lateritic sand, sandy clays and clayey soils with an average resistivity of 330.2 Ω-m and thickness of 1.4 m. This layer is usually dry and on termite mounds and allows high infiltration of surface water due to development of macro-pores. The second layer is made up of moderately weathered schist or gneisses and has a mean resistivity of 67.6 Ω-m. This layer with an average thickness of 10.5 m is saturated and represents a major aquifer unit. The third layer is a highly resistive layer with average resistivity of 30966.7 Ω-m. It represents the fractured/fresh bedrock with an average depth to the top of the layer being 11.93 m. Hand dug wells and shallow hand pumps abstract groundwater from the second layer while deep boreholes can pierce into the fractured/fresh bedrock.

#### 3.2.2. Four-Layer Model

The four-layer curve is the dominant curve model with about 68% of sounding result falling into this category. The first layer has an average resistivity of 227.4 Ω-m and average thickness of 1.16 m. It is composed of highly weathered surficial material including lateritic sandy clays. The second layer is characterized by fairly high average resistivity of 1117.8 Ω-m and thin layer thickness of 2.08 m. The third layer represents a slightly weathered layer of schist and gneissic rocks with moderate average resistivity of 572.8 Ω-m and mean thickness of 11.56 m. The fourth layer is the fractured/fresh bedrock composed of high average resistivity of 70187.3 Ω-m. The average depth to the top of this layer is 14.8 m. Abstraction of groundwater here is through hand-dug wells for layer two (dry to slightly saturated), shallow hand pumps and motorized boreholes in layer three (slightly to highly saturated), and deep boreholes into the fractured bedrock of layer four.

#### 3.2.3. Five-Layer Model

Only 11% of curves depict five-layer models. The first layer of this model is characterized by an average resistivity of 327 Ω-m and a mean thickness of 1.01 m. The second layer is a highly weathered layer with low average resistivity value of 82.8 Ω-m and average thickness of 1.29 m. The third layer is composed of moderately weathered schist and gneissic rocks with high average resistivity of 3853 Ω-m and mean thickness of 1.34 m. The fourth layer of this model represents a slightly weathered/fractured layer with average resistivity of 155.6 Ω-m and mean thickness of 9.66 m. The first three layers are dry or slightly saturated, where groundwater abstraction is mainly by shallow hand dug wells that are productive only during wet periods. The fourth layer makes up the major regolith aquifer and groundwater abstraction is through hand-pumps and motorized boreholes.

### 3.3. Geo-Electric Layer Correlations with Well-Logs

**Figure 5:**
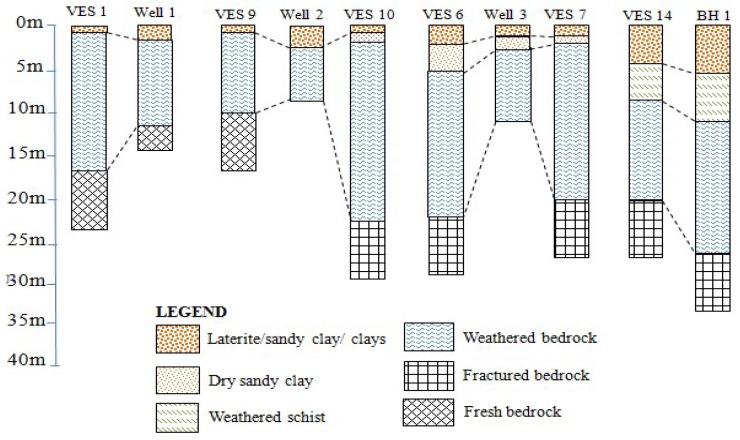
Well and borehole logs correlated with VES interpretation results.

The interpretation of VES data can be ambiguous and only when adequate knowledge of surface geology and established good controls through well-logs are available can a reliable interpretation be established (e.g., [40,72,73]). For this reason, three termite mounds/VES points were located in close proximity to dug-wells and one other to a deep borehole. Analysis of the wells and borehole lithological logs were used as constraints to guide the VES interpretation. Good correlations were found between the litho-logs and the VES interpreted results (Figure 5).

VES 1 was interpreted from the litho-log of Well 1. It is interpreted to have three layers, the first layer being composed of a thin layer (1.5 m) of lateritic sandy to pebbly surficial material. A thick layer of weathered schist and veins of quartz, which constitutes the main regolith aquifer, characterize the second layer. The water table is at the top of this layer with low fluctuation and does not dry up even during long dry periods. The third layer is the fractured/fresh basement rock.

VES 9 and 10 were interpreted from the logs of Well 2. It showed a thin regolith first layer composed of sandy clays and a relatively thick second layer characterized by highly weathered gneissic rocks. The water table can rise to the first layer and reach a few centimeters from the ground surface during wet periods but falls drastically during dry season. The third layer corresponds to the fresh gneissic basement rock with high resistivity values.

The litho-log of Well 3 aided the interpretation of VES 6 and 7. The interpretation showed a thin and moist clayey layer representing the first layer and another brighter layer of relatively dry sandy clay that represented the second layer. The third layer is composed of thick layer of weathered gneissic rock that is saturated with groundwater. The water table here aligns with the third layer but is prone to seasonal variation. The fourth layer, with a depth of about 20 m with respect to the ground surface, is characterized by fractured gneissic basement rock from where deep boreholes abstract groundwater.

Borehole 1 was drilled on VES 14 and litho-log from the borehole was used to interpret VES 15 as well. The interpretation indicated a first layer of about 5.3 m thickness to be composed of mottled clays (highly weathered schist with dark shiny speckles of muscovite mica) followed by a second layer of relatively equal thickness but composed of moderately weathered schist and slightly saturated with groundwater. The third layer with a thickness of about 16 m is slightly weathered and fairly saturated. The fourth layer that represents the fractured basement, is about 27 m deep with respect to the ground surface and is highly saturated with groundwater. Hand dug wells in this area are not adequately productive, as they do not pierce into the main aquifer.

### 3.4. Aquifer Potential and Its Spatial Distribution

**Figure 6:**
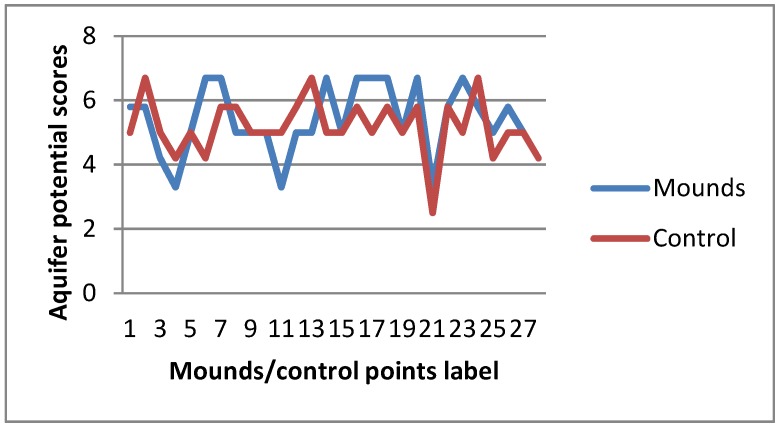
Aquifer potential around termite mounds and control areas.

**Figure 7:**
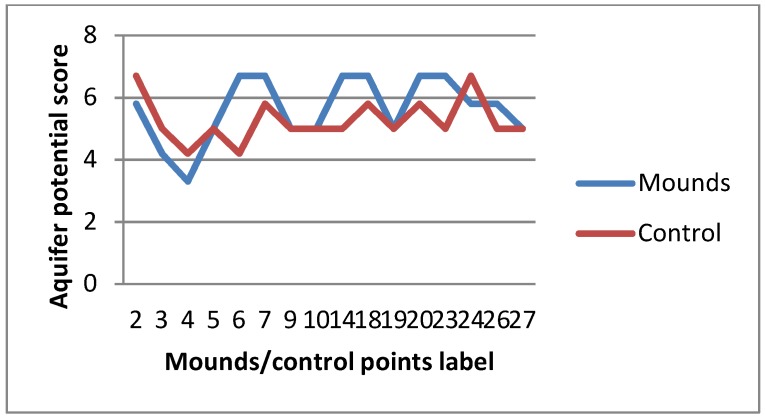
Aquifer potential around *Macrotermes* mounds and control areas.

**Figure 8:**
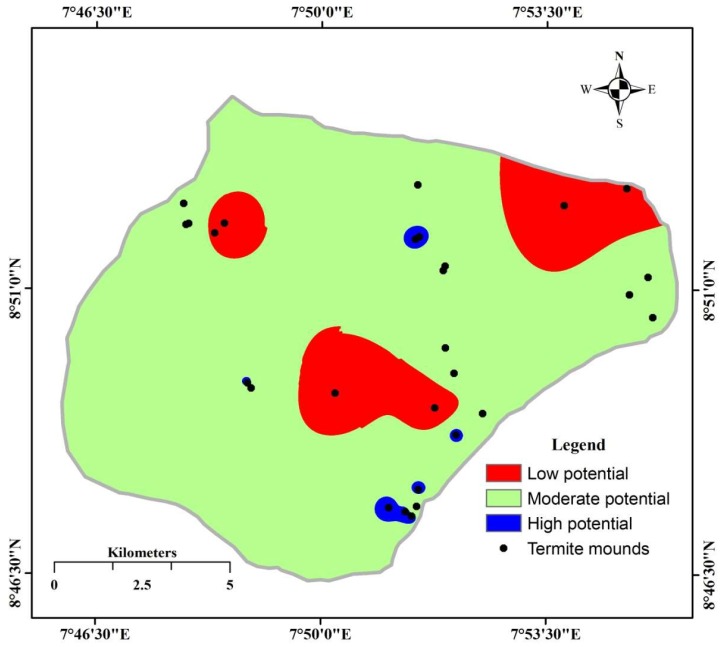
Spatial distribution of aquifer potential in Keffi, Nigeria.

The computed weighted mean derived from the three geo-electrical parameters (Table S5) generally revealed aquifers in the study area as having low to moderate groundwater potential (Figure 6, Figure 7 and Figure 8). This result is slightly different from the work of Anudu et al. [26], which obtained few VES points with high aquifer potential. The high potential VES points were obtained around the Dadin Kowa area, where there are no visible termite activities, hence our investigation did not extend to that area.

From the result, about 43% of termite mound sites have greater aquifer potential than adjacent control areas, while only about 28.5% of the mound sites indicated the contrary. This represents about 43% greater chances of success should groundwater structures be sited around termite mounds than surrounding areas. One of the primary reasons of enhanced aquifer potential around termite mounds is observed to be the thickness of the weathered layer (saprolite). Termites either have the ability to locate places with deeper weathering horizon or are themselves agents of biological weathering. Mounds of the genus *Macrotermes*, which have the largest body and colony size among other termite species [8], have only about 25% probability of poorer aquifer potentials around them compared with surrounding areas (Figure 7). Although our data for other species are very limited, they demonstrate that mounds of the genus *Nasutitermes* (smallest of the termite species) are usually indicative of promising aquifer potential. These mounds are the smallest in terms of both height and diameter in our study sites and, although of less ecological significance compared with larger mounds [74], they increase in size over time [75].

There is no visible influence of the activeness or otherwise of mounds on aquifer potential. This is probably because the original builders and/or later colonists have destroyed the crust below during the active periods of the mounds. The destroyed crust would likely remain long after the mound occupants have deserted [4]. Termite mounds without canopy protection are more likely to indicate good aquifer potential compared to those under tree canopies. However, this likelihood is not statistically significant (*U* = 69.5, *p* = 0.579). Based on the 28 VES points from termite mound sites, the aquifer potential map was prepared. Weighted mean potential values ranged from 3.3 to 6.7 and where thus categorized into 3 classes of 3.30–5.00, 5.01–6.50 and 6.51–6.70 for low potential, moderate potential, and high potential, respectively.

The aquifer potential map produced shows that the study area is dominated by moderate potential aquifers covering an area of about 133.8 km^2^, while poor potential aquifers are restricted to the northeastern and central parts covering a total area of about 21.5 km^2^. There are very few isolated patches with high aquifer potential towards the northern and southern parts with a total area of only about 1.2 km^2^. Groundwater development in this region should be preferably on a small scale due to the lack of adequate high potential aquifers.

## 4. Conclusions

Weathered and fractured bedrocks constitute the main aquifer unit of the study area and their spatial distribution is, of course, erratic. Locating groundwater structures in this environment requires a thorough investigation to lead to success. Physical and hydraulic measurements around termite mounds established significantly higher rates of surface water infiltration and lower soil water content. Since the main source of aquifer recharge in the study area is infiltration from rainwater, locations of termite mounds can be an indication by proxy of prospective groundwater reserves. It can be deduced that locations of termite mounds are preferential sites for surface water drainage and are consequently good for groundwater storage at depths. Mean weights derived from VES parameters indicated that the aquifers beneath termite mounds tend to have greater potentials relative to the surrounding areas. The source of groundwater in the study area exists within weathered/fractured layers with water table ranging from about 1–13 m from the surface. The groundwater potential is generally moderate to low, with few isolated places having high potential; thus, the area is suitable for small-scale groundwater development schemes. These findings should be of interest to groundwater managers as it provides a contextual guide for groundwater development in the study area.

## Figures and Tables

**Table 1:** Summary of physical characteristics of surveyed termite mounds.

Mound ID	Height (m)	Diameter (m)	Status	Species	Under Canopy?
TM-1	1.2	2.64	Not active	-	No
TM-2	0.34	1.35	Active	*Macrotermes*	Yes
TM-3	1.67	3.21	Active	*Macrotermes*	Yes
TM-4	1.7	2.17	Active	*Macrotermes*	Yes
TM-5	2.03	1.8	Active	*Macrotermes*	Yes
TM-6	2.07	2.6	Active	*Macrotermes*	Yes
TM-7	0.8	3.65	Active	*Macrotermes*	Yes
TM-8	2.5	1.45	Not active	*-*	Yes
TM-9	1.22	2.71	Active	*Macrotermes*	No
TM-10	0.77	2.3	Active	*Macrotermes*	No
TM-11	0.95	1.3	Active	*Coptotermes*	Yes
TM-12	0.4	0.9	Not active	*-*	Yes
TM-13	1.72	1.55	Not active	*-*	Yes
TM-14	1.45	1.95	Active	*Macrotermes*	Yes
TM-15	1.3	2.6	Active	*Coptotermes*	Yes
TM-16	0.32	0.43	Active	*Nasutitermes*	Yes
TM-17	0.42	1.03	Not active	*Nasutitermes*	Yes
TM-18	0.64	2.6	Active	*Macrotermes*	Yes
TM-19	0.77	2.1	Active	*Macrotermes*	No
TM-20	1.62	2.38	Active	*Macrotermes*	Yes
TM-21	0.3	0.9	Not active	-	No
TM-22	1.4	1.97	Not active	-	No
TM-23	0.98	1.07	Active	*Macrotermes*	Yes
TM-24	0.8	4.52	Active	*Macrotermes*	No
TM-25	2.77	1.67	Not active	*-*	Yes
TM-26	1.34	2.45	Active	*Macrotermes*	No
TM-27	2.65	1.8	Active	*Macrotermes*	Yes
TM-28	3.1	2.55	Not active	-	Yes

**Table 2:** Aquifer potential as a function of depth to bedrock, weathered bedrock resistivity and fractured bedrock resistivity.

Theme	Depth (m)	Resistivity (Ohm-m)	Aquifer Characteristics	Weighting
Depth to bedrock	<10		Thin regolith	2.5
	10–20		Medium regolith	5.0
	20–30		Optimum weathering	7.5
	>30		Deep weathering	10.0
Weathered bedrock resistivity		<20	Clays with limited potential	7.5
		21–100	Optimum weathering	10.0
		101–150	Medium weathering condition	7.5
		151–300	Little weathering	5.0
		>300	Negligible potential	2.5
Fractured bedrock resistivity		<750	High fracture, high potential	10.0
		750–1500	Medium potential	7.5
		1500–3000	Low potential	5.0
		>3000	Little or no weathering	2.5

Source: [26,29,53,54].

**Table 3:** Mean or mean rank of physical and hydraulic properties of termite mounds and adjacent control soils.

Location	Particle Size Distribution			Bulk Density (g/cm^3^)	Porosity (%)	Infiltration Rate (mm/s)	Soil Water Content (%)
	Sand (%)	Silt (%)	Clay (%)				
Termite mounds	58.20 *	26.01 *	38.80 *	1.93	27.3	39.36 *	10.43 *
Control soils	76.64 *	16.92 *	18.20 *	1.94	26.7	17.64 *	13.03 *

Asterisks (*) indicate significant difference at *p* ≤ 0.05.

**Table 4:** Average resistivity and thicknesses of geo-electric layers beneath termite mounds and control areas.

No. of Layers		Layer Resistivity (Ω-m)					Layer Thickness (m)				Regolith Thickness (m)
		ρ1	ρ2	ρ3	ρ4	ρ5	h1	h2	h3	h4	
**3**	a	3.3 × 10^2^	6.7 × 10^1^	3.0 × 10^4^	-	-	1.4	10.5	-	-	11.9
	b	3.9 × 10^2^	5.8 × 10^1^	3.3x 10^4^	-	-	1.3	4.1	-	-	5.3
4	a	2.3 × 10^2^	1.1 × 10^3^	5.7 × 10^2^	4.7 × 10^4^	-	1.2	2.1	11.6	-	14.8
	b	5.1 × 10^2^	8.3 × 10^2^	1.7 × 10^3^	2.9 × 10^4^	-	0.5	1.9	10.1	-	12.5
5	a	3.3 × 10^2^	8.3 × 10^1^	3.8 × 10^3^	1.6 × 10^2^	8.0 × 10^4^ *	1.0	1.3	1.3	9.7	13.3
	b	4.8 × 10^2^	1.3 × 10^4^	6.5 × 10^2^	3.3 × 10^2^	1.6 × 10^4^ *	0.6	0.9	3.1	5.3	9.8

a = Termite mounds, b = Control areas, ρ = layer resistivity and h = layer thickness. * indicate significant difference at *p* ≤ 0.05.

## Supplementary Materials

The following are available online at https://www.mdpi.com/1424-8220/19/9/2107/s1, Figure S1: Soil texture classification chart indicating the soil types in the study area, Table S1: Soil texture of mounds and control area soils, Table S2: Result of bulk density and porosity measurements on mounds and control soils, Table S3: Result of soil water content in mounds and control area soils, Table S4:Result of infiltration test on termite mounds and control areas, Table S5: Resistivity and layer thicknesses beneath termite mounds and control areas.

## References

[1] Dangerfield J.M., Mccarthy T.S., Ellery W.N. (1998). The mound-building termite Macrotermes michaelseni as an ecosystem engineer. J. Trop. Ecol..

[2] Bignell D.E., Eggleton P., Abe T., Bignell D.E., Higashi H. (2000). Termites in ecosystem. Termites: Evolution, Sociality, Symbiosis, Ecology.

[3] Bottinelli N., Jouquet P., Capowiez Y., Podwojewski P., Grimaldi M., Peng X. (2015). Why is the influence of soil macrofauna on soil structure only considered by soil ecologists?. Soil Tillage Res..

[4] Léonard J., Rajot J.L. (2001). Influence of termites on runoff and infiltration: Quantification and analysis. Geoderma.

[5] Choosai C., Mathieu J., Hanboonsong Y., Jouquet P. (2009). Termite mounds and dykes are biodiversity refuges in paddy fields in north-eastern Thailand. Environ. Conserv..

[6] Jouquet P., Traoré S., Choosai C., Hartmann C., Bignell D. (2011). Influence of termites on ecosystem functioning. Ecosystem services provided by termites. Eur. J. Soil Biol..

[7] Moura E.G., das CF Aguiar A., Piedade A.R., Rousseau G.X. (2014). Contribution of legume tree residues and macrofauna to the improvement of abiotic soil properties in the eastern Amazon. Appl. Soil Ecol..

[8] Turner J.S., D’Odorico P., Porporato A. (2006). Termites as mediators of the water economy of Arid Savanna ecosystems. Dryland Ecohydrology.

[9] Davies A.B., Baldeck C.A., Asner G.P. (2016). Termite mounds alter the spatial distribution of African savanna tree species. J. Biogeogr..

[10] Lynch D.K., Jordan F., Reynolds E.R. (2013). Vegetation Lineaments Near Pearblossom, CA. Raising Questions in the Central Mojave Desert, Proceedings of the 2013 Desert Symposium, California, USA, 18–21 April 2013.

[11] Ferriz H., Bizuneh G. Development and management of water resources. Proceedings of the Ethio-Forum ESRDF: Community Driven Poverty Eradication and Restorative Development in Ethiopia.

[12] West W.F. (1965). Some unconventional ideas on prospecting. Chamb. Mines J..

[13] Watson J.P. (1972). The distribution of gold in termite mounds and soils at a gold anomaly in Kalahari sand. Soil Sci..

[14] Davies A.B., Levick S.R., Asner G.P., Robertson M.P., Van Rensburg B.J., Parr C.L. (2014). Spatial variability and abiotic determinants of termite mounds throughout a savanna catchment. Ecography.

[15] Ahmed II J.B., Pradhan B. (2018). Termite Mounds as Bio-Indicators of Groundwater: Prospects and Constraints. Pertan. J. Sci. Technol..

[16] Mège D., Rango T. (2010). Permanent groundwater storage in basaltic dyke fractures and termite mound viability. J. Afr. Earth Sci..

[17] Bargués Tobella A., Reese H., Almaw A., Bayala J., Malmer A., Laudon H., Ilstedt U. (2014). The effect of trees on preferential flow and soil infiltrability in an agroforestry parkland in semiarid Burkina Faso. Water Resour. Res..

[18] West W.F. (1970). Termite Prospecting. Chamb. Mines J..

[19] Safriel U., Adeel Z., Niemeijer D., Puigdefabregas J., White R., Lal R., Winslow M., Ziedler J., Prince S., Archer E., Hassan R., Scholes R., Ash N. (2005). Dryland Systems. Ecosystems and Human Well-Being: Current State and Trends: Findings of the Condition and Trends Working Group.

[20] Bosch C., Hommann K., Rubio G.M., Sadoff C., Travers L. (2001). Water, Sanitation and Poverty.

[21] Liu W., Zhao M., Xu T. (2018). Water Poverty in Rural Communities of Arid Areas in China. Water.

[22] Dowuona G.N.N., Atwere P., Dubbin W., Nude P.M., Mutala B.E., Nartey E.K., Heck R.J. (2012). Characteristics of termite mounds and associated Acrisols in the coastal savanna zone of Ghana and impact on hydraulic conductivity. Nat. Sci..

[23] Peel M.C., Finlayson B.L., Mcmahon T.A. (2007). Updated world map of the Koppen-Geiger climate classification. Hydrol. Earth Syst. Sci..

[24] Lyam A., Mamman A.B., Oyebaji J.O., Peters S.W. (2000). Nasarawa State. Nigeria: Survey of States.

[25] Mahmud A., Achide A.S. (2012). Analysis of Land use/Land cover changes to monitor urban sprawl in Keffi-Nigeria. Environ.Res. J..

[26] Anudu G.K., Essien B.I., Obrike S.E. (2014). Hydrogeophysical investigation and estimation of groundwater potentials of the Lower Palaeozoic to Precambrian crystalline basement rocks in Keffi area, north-central Nigeria, using resistivity methods. Arab. J. Geosci..

[27] Obaje N.G., Nzegbuna A.I., Moumouni A., Ukaonu C.E. (2005). Geology and Mineral Resources of Nasarawa State.

[28] Obaje N.G. (2009). Geology and Mineral Resources of Nigeria.

[29] Jatau B.S., Bajeh I. (2007). Hydrogeological appraisal of parts of Jemaa Local Government Area, North-Central Kaduna State, Nigeria. Res. J. Appl. Sci..

[30] Adeyemo I.A., Omosuyi G.O., Ojo B.T., Adekunle A. (2017). Groundwater Potential Evaluation in a Typical Basement Complex Environment Using GRT Index—A Case Study of Ipinsa-Okeodu Area. J. Geosci. Environ. Prot..

[31] Bayewu O.O., Oloruntola M.O., Mosuro G.O., Laniyan T.A., Ariyo S.O., Fatoba J.O. (2017). Geophysical evaluation of groundwater potential in part of southwestern Basement Complex terrain of Nigeria. Appl. Water Sci..

[32] Edet A.E., Okereke C.S. (2002). Delineation of shallow groundwater aquifers in the coastal plain sands of Calabar area (Southern Nigeria) using surface resistivity and hydrogeological data. J. Afr. Earth Sci..

[33] Fenta A.A., Kifle A., Gebreyohannes T., Hailu G. (2014). Spatial analysis of groundwater potential using remote sensing and GIS-based multi-criteria evaluation in Raya Valley, northern Ethiopia. Hydrogeol. J..

[34] Naghibi S.A., Pourghasemi H.R. (2015). Comparative assessment between three machine learning models and their performance comparison by bivariate and multivariate statistical methods in groundwater potential mapping. Water Resour. Manag..

[35] Kordestani M.D., Naghibi S.A., Hashemi H., Ahmadi K., Kalantar B. (2018). Groundwater potential mapping using a novel data-mining ensemble model. Hydrogeol. J..

[36] Beretta A.N., Silbermann A.V., Paladino L., Torres D., Bassahun D., Musselli R., García-Lamohte A. (2014). Soil texture analyses using a hydrometer: Modification of the Bouyoucos method. Cienc. Investig. Agrar..

[37] Hamzah U., Malin E., Samsudin A.R. (2007). Groundwater investigation in Kuala Selangor using vertical electrical sounding (VES) surveys. Environ. Geol..

[38] Bibby H.M., Risk G.F., Caldwell T.G., Heise W. (2009). Investigations of deep resistivity structures at the Wairakei Geothermal field. Geothermics.

[39] Gopinath G., Seralathan P. (2004). Vertical electrical soundings for delineation of groundwater prospect zone in a crystalline terrains of the muvattupuzha river basin, Kerala, India. ISH J. Hydraul. Eng..

[40] Suneetha N., Gupta G., Laxminarayana M. (2017). Evaluation of geoelectric parameters to delineate the subsurface fractures for groundwater exploration around coastal Maharashtra, India. J. Coast. Sci..

[41] Adji T.N., Sejati S.P. (2014). Identification of groundwater potential zones within an area with various geomorphological units by several field parameters and a GIS approach in Kulon Progo Regency, Java, Indonesia. Arab. J. Geosci..

[42] Alhassan U.D., Obiora D.N., Okeke F.N. (2015). The assessment of aquifer potentials and aquifer vulnerability of the assessment of aquifer potentials and aquifer vulnerability of Southern Paiko, North central Nigeria, using geoelectric method. Glob. J. Pure Appl. Sci..

[43] Panthulu T.V., Krishnaiah C., Shirke J.M. (2001). Detection of seepage paths in earth dams using self-potential and electrical resistivity methods. Eng. Geol..

[44] Caris J.P.T., Van Asch T.W.J. (1991). Geophysical, geotechnical and hydrological investigations of a small landslide in the French Alps. Eng. Geol..

[45] Larisa G., Antonina P., Anatoly P.A., Bradley J.C. (2001). Vertical Electrical Sounding Method for Agricultural Soil Survey. Application of Geophysics to Engineering and Environmental Problems, Proceedings of the 24th SAGEEP Conference, Charleston, SC, USA, 10–14 April 2011.

[46] Choudhury K., Saha D.K., Chakraborty P. (2001). Geophysical study for saline water intrusion in a coastal alluvial terrain. J. Appl. Geophys..

[47] Soupios P., Papadopoulos I., Kouli M., Georgaki I., Vallianatos F., Kokkinou E. (2007). Investigation of waste disposal areas using electrical methods: A case study from Chania. Environ. Geol..

[48] Zohdy A.A.R., Eaton G.P., Mabey D.R. (1974). Application of surface geophysics to groundwater investigations. USGS-TWRA, Book 2.

[49] Alhassan U.D., Obiora D.N., Okeke F.N. (2017). Geoelectrical investigation of groundwater potentials of northern Paiko, Niger State, North Central Nigeria. J. Earth Sci..

[50] Mogaji K.A. (2016). Geoelectrical parameter-based multivariate regression borehole yield model for predicting aquifer yield in managing groundwater resource sustainability. J. Taibah Univ. Sci..

[51] Barker R., Blunk I., Smith I. (1999). Geophysical consideration in the design of UK National Resistivity Sounding Database. First Break.

[52] Sabet M.A. (1975). Vertical Electrical Resistivity Soundings to Locate Ground Water Resources: A Feasibility Study.

[53] Olayinka A.I. (1996). Non uniqueness in the interpretation of bedrock resistivity from sounding curves and its hydrological implications. Water Resour. J. NAH.

[54] Olayinka A.I., Akpan E.J., Magbagbeola O.A. (1997). Geoelectric sounding for estimating aquifer potential in the crystalline basement area around Shaki. Water Resour. J. NAH.

[55] Mujinya B.B., Van Ranst E., Verdoodt A., Baert G., Ngongo L.M. (2010). Termite bioturbation effects on electro-chemical properties of Ferralsols in the Upper Katanga (D.R. Congo). Geoderma.

[56] Jouquet P., Bottinelli N., Shanbhag R.R., Bourguignon T., Traoré S., Abbasi S.A. (2016). Termites: The neglected soil engineers of tropical soils. Soil Sci..

[57] Adhikary N., Erens H., Weemaels L., Deweer E., Mees F., Mujinya B.B., Baert G., Boeckx P., Van Ranst E. (2016). Effects of Spreading Out Termite Mound Material on Ferralsol Fertility, Katanga, D.R. Congo. Commun. Soil Sci. Plant Anal..

[58] Roose-Amsaleg C., Mora P., Harry M. (2005). Physical, chemical and phosphatase activities characteristics in soil-feeding termite nests and tropical rainforest soils. Soil Biol. Biochem..

[59] Seymour C.L., Joseph G.S., Makumbe M., Cumming G.S., Mahlangu Z., Cumming D.H.M. (2016). Woody species composition in an African savanna: Determined by centuries of termite activity but modulated by 50 years of ungulate herbivory. J. Veg. Sci..

[60] Jouquet P., Tessier D., Lepage M. (2004). The soil structural stability of termite nests: Role of clays in Macrotermes bellicosus (Isoptera, Macrotermitinae) mound soils. Eur. J. Soil Biol..

[61] Lavelle P.M., Dangerfield C., Frasgoso V., Eschnebrenner D.L., Hernadez B., Pashanari B.L., Woomer P.L., Swift M.J. (1994). The relationship between soil macrofauna and tropical soil fertility. The Biological Management of Tropical Soil Fertility.

[62] Ackerman I.L., Teixeira W.G., Riha S.J., Lehmanna J., Fernandes E.C.M. (2007). The impact of mound-building termites on surface soil properties in a secondary forest of Central Amazonia the impact of mound-building termites on surface soil. Appl. Soil Ecol..

[63] Flóvenz Ó.G., Hersir G.P., Saemundsson K., Ármannsson H., Fridriksson T., Sayigh A. (2012). Geothermal Energy Exploration Techniques. Comprehensive Renewal Energy.

[64] Nimmo J.R. (2013). Porosity and pore-size distribution. Reference Module in Earth Systems and Environmental Sciences.

[65] Traoré S., Tigabu M., Ouedraogo S.J., Boussim J.L., Guinko S., Lepage M. (2008). Macrotermes mounds as sites for tree regeneration in a Sudanian woodland (Burkina Faso). Plant Ecol..

[66] Abe S.S., Wakatsuki T. (2010). Possible influence of termites (*Macrotermes bellicosus*) on forms and composition of free sesquioxides in tropical soils. Pedobiology.

[67] Jouquet P., Barré P., Lepage M., Velde B. (2005). Impact of subterranean fungus-growing termites (Isoptera, Macrotermitiane) on chosen soil properties in a West African savanna. Biol. Fertil. Soils.

[68] Denovan S.E., Eggleton P., Dubbin W.E., Batchelder M., Dibog L. (2001). The effect of a soil-feeding termite, Cubitermes fungifaber (Isoptera: Termitidae) on soil properties: Termites may be an important source of soil microhabitat heterogeneity in tropical forests. Pedobiology.

[69] Eldridge D.J. (1994). Nests of ants and termites influence infiltration in a semiarid woodland. Pedobiology.

[70] Mando A., Stroosnijder L., Brussaard L. (1996). Effects of termites on infiltration into crusted soil. Geoderma.

[71] Naghibi S.A., Vafakhah M., Hashemi H., Pradhan B., Alavi S.J. (2018). Groundwater Augmentation through the Site Selection of Floodwater Spreading Using a Data Mining Approach (Case study: Mashhad Plain, Iran). Water.

[72] Olayinka A.I., Weller A. (1997). The inversion of geoelectrical data for hydrogeological applications in crystalline basement areas of Nigeria. J. Appl. Geophys..

[73] Anomohanran O. (2015). Hydrogeophysical and hydrogeological investigations of groundwater resources in Delta Central, Nigeria. Integr. Med. Res..

[74] Joseph G.S., Seymour C.L., Cumming G.S., Cumming D.H.M., Mahlangu Z. (2013). Termite mounds as islands: Woody plant assemblages relative to termitarium size and soil properties. J. Veg. Sci..

[75] Bourguignon T., Leponce M., Roisin Y. (2011). Are the spatio-temporal dynamics of soil-feeding termite colonies shaped by intra-specific competition?. Ecol. Entomol..

