# Supplementary material for: Aquifer Potential Assessment in Termites Manifested Locales Using Geo-Electrical and Surface Hydraulic Measurement Parameters
Source: Sensors (Basel). 2019 May 7;19(9):2107. doi: 10.3390/s19092107 (PMC6540149; doi:10.3390/s19092107)
Supplement: Supplementary file 1 [file sensors-19-02107-s001.pdf]

Supplementary materials

Table S1. Soil texture of mounds and control soils.

S/N	Mound/control ID	% Sand	% Silt	% Clay	Soil type
1	TM3	74.1	18.5	7.4	Sandy loam
	Control	74.1	24.1	1.8	Loamy fine sand
2	TM4	39.1	50.0	10.9	Silt loam
	Control	74.5	17.0	8.5	Sandy loam
3	TM1	69.2	23.1	7.7	Sandy loam
	Control	74.0	16.0	10.0	Sandy loam
4	TM2	74.1	14.8	11.1	Sandy loam
	Control	82.7	9.6	7.7	Loamy fine sand
5	TM5	52.4	26.2	21.4	Sandy clay loam
	Control	54.7	24.5	20.8	Sandy clay loam
6	TM8	61.3	27.0	11.7	Sandy loam
	Control	60.7	20.7	18.5	Sandy loam
7	TM9	45.5	30.3	24.2	Loam
	Control	80.0	16.0	4.0	Loamy fine sand
8	TM6	42.2	29.7	28.1	Clay loam
	Control	86.2	8.6	5.2	Loamy fine sand
9	TM10	72.2	20.4	7.4	Sandy loam
	Control	76.9	12.3	10.8	Sandy loam
10	TM7	53.5	21.1	25.4	Sandy clay loam
	Control	62.1	30.3	7.6	Sandy loam
11	TM15	59.1	18.2	22.7	Sandy clay loam
	Control	54.0	40.0	6.0	Sandy loam
12	TM12	55.4	29.2	15.4	Sandy loam
	Control	89.8	6.1	4.1	Fine sand
13	TM13	68.4	18.8	12.8	Sandy loam
	Control	85.2	11.5	3.3	Loamy fine sand
14	TM11	48.1	28.8	23.1	Loam
	Control	90.7	4.6	4.6	Fine sand
15	TM14	37.9	40.9	21.2	Loam
	Control	60.9	28.3	10.8	Sandy Loam
16	TM16	61.5	17.3	21.2	Sandy clay loam
	Control	78.3	18.3	3.4	Loamy fine sand
17	TM17	84.7	12.5	2.8	Loamy fine sand
	Control	75.8	21.0	3.2	Loamy fine sand
18	TM18	69.4	25.0	5.4	Sandy loam
	Control	89.5	7.0	3.5	Fine sand
19	TM19	64.1	21.8	14.1	Sandy loam
	Control	78.9	15.8	5.3	Loamy fine sand
20	TM20	54.6	25.0	20.3	Sandy clay loam
	Control	54.2	43.2	2.5	Sandy loam
21	TM21	83.0	12.8	4.3	Loamy fine sand
	Control	73.6	23.5	2.9	Loamy fine sand
22	TM22	36.7	44.4	18.9	Loam
	Control	86.7	10.8	2.5	Fine sand
23	TM23	41.1	35.6	23.3	Loam
	Control	82.2	11.0	6.8	Loamy fine sand

24	TM24	55.4	32.3	12.3	Sandy loam
	Control	85.1	8.5	6.4	Loamy fine sand
25	TM26	41.7	41.7	16.6	Loam
	Control	83.0	13.8	3.2	Loamy fine sand
26	TM28	74.9	16.7	8.4	Sandy loam
	Control	76.7	18.3	5.0	Loamy fine sand
27	TM27	63.8	13.8	22.4	Sandy clay loam
	Control	85.1	6.4	8.5	Loamy fine sand
28	TM25	46.1	32.3	21.5	Loam
	Control	90.2	6.6	3.2	Fine sand

Table S2. Result of bulk density and porosity measurements on mounds and control soils.

SN	TM No.	Bulk density (g/cm³)		Porosity %	
		Mound	Control area	Mound	Control area
**1**	TM 1	2.01	1.95	24.2	26.4
2	TM2	2.12	2.10	20.0	20.8
3	TM3	2.13	2.03	19.6	23.4
4	TM4	1.99	2.05	24.9	22.6
5	TM5	2.06	1.82	22.3	31.3
6	TM6	1.85	1.95	30.2	26.4
7	TM7	2.0	1.91	24.5	27.9
8	TM8	1.81	1.73	31.7	34.7
9	TM9	1.89	1.85	28.7	30.2
10	TM10	2.05	1.87	22.6	29.4
11	TM11	2.02	2.06	23.8	22.3
12	TM12	1.94	1.80	26.8	32.1
13	TM12	1.85	1.85	30.2	30.2
14	TM94	2.05	2.02	22.6	23.8
15	TM15	1.93	1.85	27.2	30.2
16	TM16	1.84	1.92	30.6	27.5
17	TM17	1.72	2.06	35.1	22.3
18	TM18	1.81	1.84	31.7	30.6
19	TM19	1.72	2.08	35.1	21.5
20	TM20	1.77	1.92	33.2	27.5
21	TM21	1.78	1.96	32.8	26.0
22	TM22	2.06	1.93	22.3	27.2
23	TM23	2.06	1.95	22.3	26.4
24	TM24	1.84	1.96	30.6	26.0
25	TM25	1.77	1.95	33.2	26.4
26	TM26	2.01	2.10	24.2	20.8
27	TM27	1.93	1.88	27.2	29.1
28	TM28	1.94	1.88	26.8	29.1

Table 3. Result of soil water content in mounds and control area soils.

S/N	Mound ID	Wet weight (g)	Dry weight (g)	Water content (%)
1.	TM 1	172	154	10.5
	Control	167	145	13.2
2.	TM 2	181	166	8.3
	Control	180	153	15.0
3.	TM 3	182	160	12.1
	Control	174	148	14.9

4.	TM 4	170	143	15.9
	Control	175	145	17.1
5.	TM 5	176	158	10.2
	Control	156	140	10.3
6.	TM 6	158	145	8.2
	Control	167	145	13.2
7.	TM 7	171	156	8.8
	Control	163	145	10.7
8.	TM 8	155	142	8.4
	Control	148	133	10.1
9.	TM 9	162	149	8.0
	Control	158	142	10.1
10.	TM 10	175	157	10.3
	Control	160	142	11.3
11.	TM 11	173	154	11.0
	Control	176	152	13.6
12.	TM 12	166	147	11.5
	Control	154	130	15.6
13.	TM 13	158	139	12.0
	Control	158	145	8.2
14.	TM 14	175	155	11.4
	Control	173	150	13.3
15.	TM 15	165	153	7.3
	Control	158	142	10.1
16.	TM 16	157	137	12.7
	Control	164	139	15.2
17.	TM 17	147	133	9.5
	Control	151	134	11.3
18.	TM 18	155	142	8.4
	Control	157	141	10.2
19.	TM 19	147	132	10.2
	Control	168	138	17.9
20.	TM 20	151	137	9.3
	Control	164	146	11.0
21.	TM 21	152	133	13.8
	Control	168	144	14.3
22.	TM 22	176	157	10.8
	Control	165	142	13.9
23.	TM 23	176	163	13.1
	Control	167	140	16.2
24.	TM 24	157	141	10.2
	Control	168	149	11.3
25.	TM 25	151	132	12.6
	Control	167	141	15.6
26.	TM 26	172	156	9.3
	Control	180	154	14.4
27.	TM 27	165	137	8.5
	Control	161	143	11.2
28.	TM 28	166	150	9.6
	Control	161	136	15.5

Table S4. Result of infiltration rate (mm/s) on termite mounds and control soils.

SN	TM No.	Mound	Control area
1	TM 1	0.14	0.56
2	TM2	0.02	0.10
3	TM3	2.51	0.04
4	TM4	1.06	0.01
5	TM5	1.01	0.19
6	TM6	1.59	0.18
7	TM7	1.76	0.13
8	TM8	0.34	0.06
9	TM9	2.07	0.05
10	TM10	1.64	0.08
11	TM11	3.70	0.02
12	TM12	0.92	0.02
13	TM13	2.01	0.01
14	TM14	0.42	0.01
15	TM15	0.66	0.04
16	TM16	0.02	0.05
17	TM17	0.34	0.10
18	TM18	1.36	0.04
19	TM19	1.47	0.02
20	TM20	0.02	0.08
21	TM21	1.21	0.01
22	TM22	1.24	0.24
23	TM23	3.88	0.04
24	TM24	3.36	1.58
25	TM25	0.03	0.02
26	TM26	0.03	0.02
27	TM27	0.90	0.07
28	TM28	0.86	0.05

Table S5. Resistivity and layer thicknesses around termite mounds and control areas.

Mound ID	Depth to bedrock (m)	Weathered bedrock resistivity	Fractured bedrock resistivity	Geometric mean
**TM1**	**16.83**	**70.5**	**16448**	5.8
Cont1	9.47	68.8	8210	5.0
TM2	12.01	58.7	21764	5.8
Cont2	6.05	41.7	800	6.7
TM3	15.53	260	103536	4.2
Cont3	4.42	92.6	14906	5.0
TM4	16.24	303	76853	3.3
Cont4	11.99	193	22206	4.2
TM5	4.47	43.1	60351	5.0
Cont5	2.16	27.6	87584	5.0
TM6	20.87	2707	24.5	6.7
Con6	28.32	463	19283	4.2
TM7	19.94	138	1225	6.7
Cont7	11.2	84.7	8455	5.8
TM8	8.67	66.1	42533	5.0
Cont8	10.9	32.5	21582	5.8
TM9	10.21	131	22848	5.0

Cont9	2.64	25.9	45052	5.0
TM10	23.17	399	2706	5.0
Cont10	5.74	47.2	27837	5.0
TM11	16.87	4180	67770	3.3
Cont11	4.95	34.8	64763	5.0
TM12	5.08	31.3	27262	5.0
Cont12	1.93	10.8	1080	5.8
TM13	6.07	27.4	66931	5.0
Cont13	11.77	83.1	2668	6.7
TM14	21.24	2041	10.4	6.7
Cont14	9.54	67.9	80634	5.0
TM15	4.93	19.5	16202	5.0
Cont15	4.96	21.9	17598	5.0
TM16	7.02	14.2	708	6.7
Cont16	11.03	1167	2.29	5.8
TM17	11.67	43.2	66284	6.7
Cont17	4.48	87.1	91762	5.0
TM18	23.24	71.4	16448	6.7
Cont18	37.55	166	49306	5.8
TM19	14.32	53.9	16448	5.0
Cont19	1.64	10.6	542	5.0
TM20	30.1	110	42689	6.7
Cont20	8.24	79.8	1456	5.8
TM21	10.9	364	60829	3.3
Cont21	6.67	22654	3193	2.5
TM22	9.97	58 10	44797	5.8
Cont22	13.72	62 10	28560	5.8
TM23	28.5	37	8086	6.7
Cont23	6.48	37.1	30362	5.0
TM24	15.0	97.7	122965	5.8
Cont24	2.18	10.6	542	6.7
TM25	2.93	58.7	82890	5.0
Cont25	26.77	401	87859	4.2
TM26	16.53	138	2653	5.8
Cont26	3.49	23.8	45361	5.0
TM27	12.36	106	42041	5.0
Cont27	5.5	41.3	40347	5.0
TM28	8.13	127	303724	4.2
Cont28	13.21	486	2422	4.2

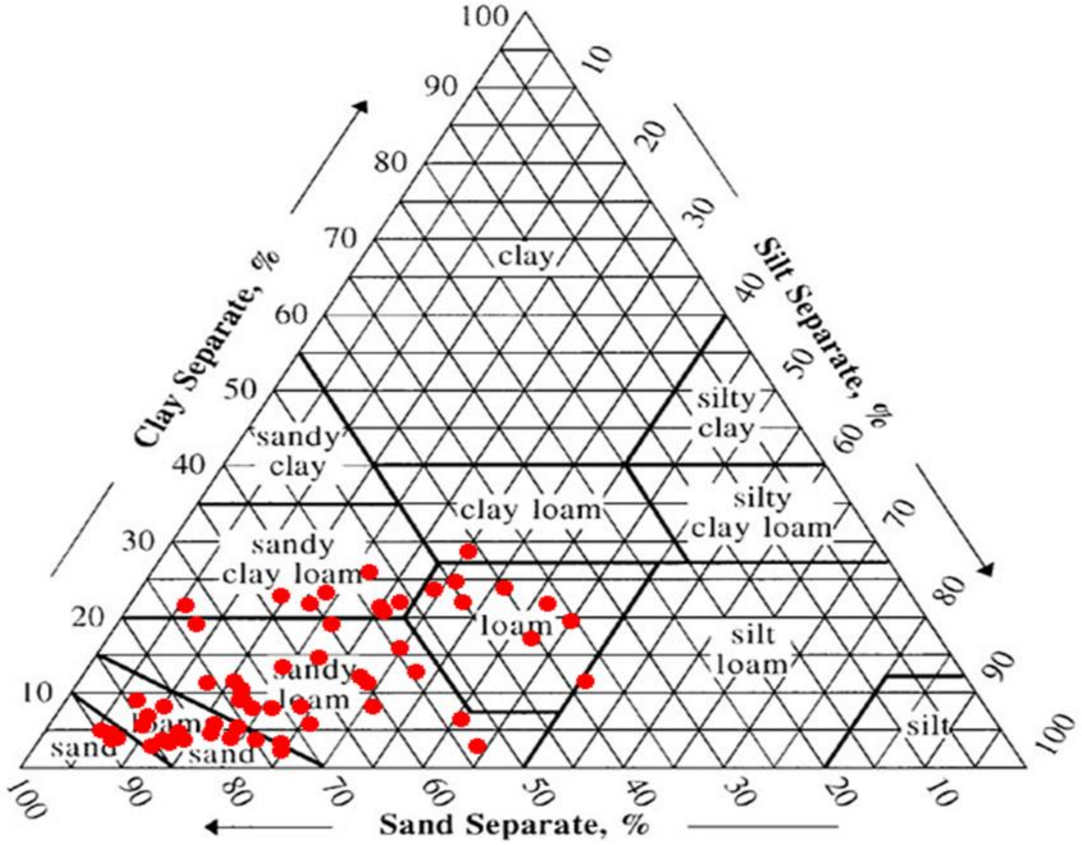

Figure S1. Soil texture classification chart indicating the soil types in the studied area (red dots).
